# Plasma extracellular vesicle delivery of miR-210-3p by targeting ATG7 to promote sepsis-induced acute lung injury by regulating autophagy and activating inflammation

**DOI:** 10.1038/s12276-021-00651-6

**Published:** 2021-07-28

**Authors:** Guang Li, Bo Wang, Xiangchao Ding, Xinghua Zhang, Jian Tang, Huiqing Lin

**Affiliations:** 1 Department of Critical Care Medicine, Renmin Hospital of Wuhan University, 430060 Wuhan, P.R. China; 2 Department of Thoracic Surgery, Renmin Hospital of Wuhan University, 430060 Wuhan, P.R. China; 3 Department of Thoracic Surgery, The First Affiliated Hospital of Nanchang University, 330006 Nanchang, P.R. China

**Keywords:** Infection, Immunological disorders

## Abstract

Extracellular vesicles (EVs) can be used for intercellular communication by facilitating the transfer of miRNAs from one cell to a recipient cell. MicroRNA (miR)-210-3p is released into the blood during sepsis, inducing cytokine production and promoting leukocyte migration. Thus, the current study aimed to elucidate the role of plasma EVs in delivering miR-210-3p in sepsis-induced acute lung injury (ALI). Plasma EVs were isolated from septic patients, after which the expression of various inflammatory factors was measured using enzyme-linked immunosorbent assay. Cell viability and apoptosis were measured via cell counting kit-8 and flow cytometry. Transendothelial resistance and fluorescein isothiocyanate fluorescence were used to measure endothelial cell permeability. Matrigel was used to examine the tubulogenesis of endothelial cells. The targeting relationship between miR-210-3p and ATG7 was assessed by dual-luciferase reporter assays. The expression of ATG7 and autophagy-related genes was determined to examine autophagic activation. A sepsis mouse model was established by cecal ligation and puncture (CLP)-induced surgery. The level of miR-210-3p was highly enriched in septic EVs. MiR-210-3p enhanced THP-1 macrophage inflammation, BEAS-2B cell apoptosis, and HLMVEC permeability while inhibiting angiogenesis and cellular activity. MiR-210-3p overexpression reduced ATG7 and LC3II/LC3I expression and increased P62 expression. Improvements in vascular density and autophagosome formation, increased ATG7 expression, and changes in the ratio of LC3II/LC3I were detected, as well as reduced P62 expression, in adenovirus-anti-miR-210-3p treated mice after CLP injury. Taken together, the key findings of the current study demonstrate that plasma EVs carrying miR-210-3p target ATG7 to regulate autophagy and inflammatory activation in a sepsis-induced ALI model.

## Sepsis: The mechanism of acute lung injury

A short non-coding microRNA carried in nanosized packets called extracellular vesicles (EVs) is a key factor in the acute lung injury (ALI) seen in sepsis, which occurs when the body’s response to infection spirals out of control, leading to organ failure. ALI is one of the most common causes of sepsis-related fatality, but how sepsis causes ALI is unclear. Huiqing Lin at Wuhan University in China and co-workers investigated the role of miR-210-3p, thought to be involved in lung function, in sepsis-induced ALI. Sampling blood-borne EVs from sepsis patients showed increased miR-210-3p levels. Further research showed that miR-210-3p hyper-activates a gene involved in degradation of damaged cells, causing excessive degradation. Inhibiting miR-210-3p in a mouse model of sepsis decreased lung injury. Targeting this microRNA shows promise for protecting lung tissue against sepsis-induced damage.

## Introduction

Sepsis is characterized by the host inflammatory response to invading pathogens triggering a potentially life-threatening hemodynamic instability, often accompanied by multiple organ dysfunction^1^. Acute lung injury (ALI) remains one of the most common causes of death in patients suffering from sepsis^2^, yet the molecular and cellular mechanisms that drive inflammation, as well as the deterioration of lung function, are not fully understood. Previous reports have indicated that Mac-1 upregulates neutrophils to inhibit pulmonary microcirculation in sepsis-induced ALI, which can be reversed by a Mac-1 inhibitor^3^. Microvascular leakage and downregulation of the gene expression of Angpt1 (Ang1), Tek (Tie2), and Kdr (Vegfr2 or Flk-1) have been implicated in sepsis-induced ALI^4^. However, the molecular and cellular mechanisms underpinning this condition fail to provide a full picture of sepsis pathogenesis. ALI is characterized by excessive autophagy and inflammation, which often contribute to elevated rates of mortality^5^. Autophagy is a process characterized by cellular engulfment of cytoplasmic proteins or organelles into vesicles, followed by fusion with lysosomes to form autophagic lysosomes and content degradation, and this process has been identified in the induction of sepsis^6^. Thus, understanding the underlying mechanism associated with autophagy and inflammation may be crucial in identifying novel treatment targets for sepsis-induced ALI.

Extracellular vesicles (EVs) are critical paracrine factors involved in intercellular communication that facilitate the transfer of miRNAs from one cell to a recipient cell^7^. The encapsulation of microRNA (miRNA or miR)-126 partially prevents lung injury in sepsis^8^. Circulating EVs in peripheral blood carrying miR-155 have been highlighted as key mediators of septic lung injury^9^. However, the role of miR-210-3p in the pathogenesis of sepsis-induced ALI has not been completely identified. MiR-210-3p has been highlighted as an oncogenic miRNA, and studies suggest that miR-210-3p is involved in various malignancies, as well as in cancer progression and metastasis^10^. MiR-210-3p is released into the blood during sepsis and induces cytokine production and leukocyte migration^11^. Dysregulation of miR-210-3p has been thought to be an important factor in lung development and function^12^. Therefore, miR-210-3p may be associated with the pathogenesis of sepsis-induced ALI, warranting further investigation. Autophagy-related 7 (ATG7) is an essential autophagy gene that encodes the E1 enzyme that is necessary for autophagosome formation^13^. ATG7 was predicted to be a target gene of miR-210-3p through our bioinformatics analysis. Thus, we hypothesized that miR-210-3p regulates autophagy and contributes to the activation of inflammation in sepsis-induced ALI. The central objective of the study was to investigate the role of plasma EV-mediated miR-210-3p delivery in autophagy and inflammatory activation in sepsis-induced ALI in vitro and in vivo.

## Methods

### Ethics statement

The current study protocol was performed with the approval of the Ethics Committee of Renmin Hospital of Wuhan University. All study subjects submitted signed written informed consent documentation prior to enrollment. All animal studies were performed in strict adherence with the standards of *The Guide for the Care and Use of Laboratory Animals* published by the National Institutes of Health. Animal experiment protocols were reviewed and approved by the Animal Ethics Committee of Renmin Hospital of Wuhan University.

### Study subjects

Clinical data were collected from 55 patients (28 males and 27 females) diagnosed with sepsis at the Renmin Hospital of Wuhan University between March 2018 and September 2019. All patients met the following inclusion criteria^14^: patients diagnosed with sepsis with highly infected sites were confirmed by etiology, and all subjects submitted informed consent and complete clinical data. All patients with blood system diseases, blood coagulation disorders, or mental illness or pregnant women were excluded from the study. An additional 30 healthy patients were recruited for this study as the control subjects (16 males and 14 females), all of whom met all inclusion and exclusion criteria at the Renmin Hospital of Wuhan University.

Sepsis was diagnosed in accordance with the *International Guidelines for Management of Sepsis and Septic Shock in 2016* as follows^14^: body temperature <36 °C or >38 °C, >20 breaths per minute, arterial partial pressure of carbon dioxide (PaCO_2_) <32 mmHg, peripheral blood leukocyte count <4 × 10^9^/L or >12 × 10^9^/L, and/or immature neutrophil levels >10%. Patients who fulfilled ≥2 of these criteria were diagnosed with the proposed conditions.

ALI was diagnosed based on the following criteria: specific primary disease or risk factors; sudden onset of illness, accompanied by dyspnea; double pulmonary infiltration suggested by chest radiograph; pulmonary artery wedge depression <18 mmHg without left atrial hypertension; and hypoxemia with an oxygenation index <300.

Additionally, Acute Physiological Assessment and Chronic Health Evaluation (APACHE) II scores and the Sepsis-related Organ Failure Assessment (SOFA) scoring system were applied to all study subjects to measure the severity of sepsis.

### EV isolation from human plasma samples

Fasting peripheral venous blood (20 mL) was collected in the morning from both healthy individuals and septic patients and subsequently placed into sodium citrate tubes for anticoagulation. Plasma EVs were obtained by cryogenic density gradient centrifugation. Following two rounds of centrifugation at 3000 × *g* for 15 min, the plasma was collected by separating the pellets, which contained blood cells and platelets, from the supernatant. The resulting plasma samples were immediately diluted 1:1 in ice-cold phosphate-buffered saline (PBS) and centrifuged at 10,000 × *g* for 30 min at 4 °C to pellet and remove EVs and microparticles. The supernatant was filtered through a 0.22-μm pore polyether sulfone (PES) filter (Millipore, Billerica, MA, USA). The clarified supernatant was then subjected to ultracentrifugation at 110,000 × *g* for 70 min at 4 °C in a SW 32 Ti Swinging Bucket rotor (*k* factor of 204, Beckman Coulter, Fullerton, CA, USA) to sediment EVs. Crude EV pellets (P120) were resuspended in ice-cold PBS, after which they were subjected to ultracentrifugation at 110,000 × *g* for 70 min. The washed pellet was resuspended in ice-cold PBS. Crude plasma EV samples were further purified via high-resolution iodixanol density gradient fractionation for subsequent experiments. At no time during the process was the plasma or plasma EVs exposed to temperatures <4 °C.

Mouse blood was collected from the orbital sinus and stored at 4 °C for 2 h. The plasma was separated from hemocytes and platelets by centrifugation at 3000 × *g* for 15 min. Next, large vesicles were removed from the plasma by centrifugation at 10,000 × *g* for 30 min at 4 °C. The supernatant was filtered through a 0.22-μm pore PES filter (Millipore). Clarified supernatants were subjected to ultracentrifugation at 110,000 × *g* for 70 min at 4 °C. The washed pellet was resuspended in ice-cold PBS and further purified by high-resolution iodixanol density gradient fractionation for subsequent use.

### Particle size and concentration measurement by nanoparticle tracking analysis (NTA)

Samples in solution were analyzed by nanoparticle tracking using a NanoSight LM10 system (NanoSight Ltd, Amesbury, UK) and NTA software (version 2.3, build 0006 beta 2). In brief, 10–20 μL of EVs were diluted in 1 mL of particle-free PBS, and a 1-mL EV sample was slowly injected into the sample tank using a 1-mL syringe in a manual fashion. After optimization, identical settings were used between measurements for the session. The main engine knob adjusted the focal length to clear the “white light spot,” followed by recording of the adjustment gain. Six videos with durations of 30 s, with a 10-s delay between measurements, were recorded for each independent replicate. Software was used to evaluate the motion track of each EV in the picture, and the diameter and concentration of EVs were automatically converted based on the Brownian motion principle, and the original concentration was converted based on the dilution ratio.

### Transmission electron microscopy (TEM)

Morphological observation was performed by TEM using a 20-μL EV suspension, which was absorbed onto formvar carbon-coated copper electron microscopy grids (200 mesh) at room temperature for 2 min, after which the samples were stained with 2% uranyl acetate for an additional minute. The grids were washed three times with PBS and maintained in a semidry state prior to observation by TEM (H-7650, Hitachi High Technologies Corporation, Tokyo, Japan) at 80 kV.

### Western blotting

Total proteins were isolated from cells and EVs using radio-immunoprecipitation assay buffer containing phosphatase inhibitors, protease inhibitors, and phenylmethylsulfonyl fluoride. Protein concentrations were determined by bicinchoninic acid assays, and the proteins were separated by 8–12% sodium dodecyl sulfate polyacrylamide gel electrophoresis and electrotransferred to 0.22-μm methanol-treated polyvinylidene difluoride membranes. The membranes were blocked with 5% nonfat milk in Tris-buffered saline with Tween 20 for 1 h at room temperature and incubated with antibodies against CD63 (1:500, ab134045, Abcam, Cambridge, UK), tumor susceptibility gene 101 (TSG101; 1:500, ab125011, Abcam), Alix (1 µg/mL, 1:1000, ab76608, Abcam), ATG7 (1:1000, #8558, Cell Signaling Technologies [CST], Beverly, MA, USA), sequestosome 1/P62 (1:1000, #39749, CST), light chain 3B (1:1000, #2775, CST), glyceraldehyde-3-phosphate dehydrogenase (GAPDH; 1:1000, #5174, CST), caspase1 (1:1000, #3866, CST), interleukin 1β (1:1000, #12242, CST), nucleotide-binding domain, leucine-rich-repeat-containing (NLR) family, pyrin domain-containing 3 (NLRP3, 1:1000, #15101, CST), and NLR family CARD domain containing 4 (NLRC4, 1:1000, #12421, CST) overnight at 4 °C, followed by incubation with horseradish peroxidase-conjugated secondary antibodies (#111035003, Jackson ImmunoResearch, West Grove, PA, USA). Color development was performed using an electrogenerated chemiluminescence system. The gray value of the corresponding protein band relative to the gray value of the GAPDH protein band was regarded as the relative protein level. The experiment was repeated three times.

### Cell culture

THP-1 cells (TIB-202, American Type Culture Collection [ATCC], Manassas, VA, USA) were cultured in 1640 medium (Gibco, Carlsbad, CA, USA) supplemented with 10% fetal bovine serum (FBS) and 1% penicillin–streptomycin (Gibco) at 37 °C, 5% CO_2_, and 95% humidity (Thermo Fisher, Austin, TX, USA). The medium was replaced with fresh medium every 3–4 days. The cells were collected for passage with a pipette without trypsinization due to their suspension properties. THP-1 cells were subsequently treated with 100 ng/mL phorbol 12-myristate 13-acetate to induce cell adhesion to the surface and macrophage differentiation for further experiments.

The human bronchial epithelium cell line (BEAS-2B cells, # CRL-9609; ATCC) was cultured in Dulbecco’s Modified Eagle Medium/F12 (Gibco) supplemented with 10% FBS (Gibco) and 1% penicillin–streptomycin (Gibco) at 37 °C, 5% CO_2_, and 95% humidity.

Human lung microvascular endothelial cells (HLMVECs, #540–05a; Cell Applications Inc., San Diego, CA, USA) were cultured in EBM-2 (containing endothelial growth factor, CC3162, Lonza, Walkersville, MD, USA) supplemented with 5% FBS (Gibco) at 37 °C, 5% CO_2_, and 95% humidity and passaged every 3–5 days. P4–P7 cells were used in the next phase of the experiment.

### Adenovirus construction

Based on the instructions of the Adenovirus Infection Agent Kit (Weizhen company, Shandong, China), THP-1 cells, BEAS-2B cells, or HLMVECs were seeded in 6-well plates at 6.0 × 10^5^ cells per well. All vectors were constructed into adenoviral plasmids by means of recombination with pAdEasy-1 in BJ5183 *Escherichia coli*. After PacI (New England Biolabs, Ipswich, MA, USA) digestion, HEK293 cells were infected with adenoviral plasmids to generate infectious adenovirus particles. Ad-NC and Ad-anti-miR-210–3p adenovirus particles (multiplicity of infection = 50) were used to infect the cells in experiments and were cultured for 72 h at 37 °C and 5% CO_2_.

### Cell transfection

According to the instructions of the Lipofectamine 3000 (Gibco) Transfection Kit, cells in the logarithmic growth phase were seeded in a 10-cm dish at 5 × 10^5^ per well and cultured at 37 °C and 5% CO_2_. After the cells reached 70–80% confluence, 25 pmol of miR-210-3p mimic, miR-210-3p inhibitor, mimic-negative control (NC), and inhibitor-NC (GenePharma, Suzhou, China) and 10 μL of transfection reagents were added to each plate. The final concentration was confirmed to be 10 pmol/mL, after which gentle shaking was performed during the transfection process. Each experiment in every group was repeated three times. The cells were cultured continuously for 48 h for follow-up experiments. In the follow-up experiment, 5 mM 3-methyladenine (3-MA) was added and incubated for 36 h for autophagy inhibition.

### Cell viability

Cells exhibiting logarithmic growth were seeded in 96-well plates at 5 × 10^3^ cells per well (100 μL/well). According to the aforementioned transfection and adenovirus infection experiment, 20 μg of plasma EVs from healthy subjects or septic patients were added to the cells, after which serum-free medium containing 10 μL of cell counting kit (CCK)-8 (CK-04, Dojindo, Kumamoto, Japan) was also added to the cells and incubated for 4 h in the dark. The absorbance value was measured using an enzyme-linked immune detector at a wavelength of 450 nm.

### Cellular uptake of EVs

Purified human plasma EVs were labeled with a PKH67 Green Fluorescent Kit (Sigma Aldrich, St Louis, MO, USA). The EVs were resuspended in 1 mL of Diluent C solution, and 4 μL of PKH-67 ethanol dye solution was added to 1 mL of diluent C to prepare a 4 × 10^−6^ M dye solution. Next, 1 mL of the EV suspension was mixed with the dye solution for 5 min, after which 2 mL of 1% bovine serum albumin was added and incubated for 1 min to terminate staining. The labeled EVs were centrifuged at 100,000 × *g* for 70 min, washed with PBS, centrifuged again, and resuspended in 50 μL of PBS. PKH67-labeled EVs were incubated with BEAS-2B cells, THP-1 cells, and HLMVECs at 37 °C for 12 h. The cells were fixed with 4% paraformaldehyde. The cells was labeled with Phalloidin-IFLuor 647 Reagent (1:1000, ab176759) and Phalloidin-IFLuor 6, a47 (Abcam) for 30 min at room temperature. The nuclei were stained with 4′,6-diamino-2-phenylindole (DAPI). EV uptake was observed under a confocal microscope (Zeiss, Thornwood, NY, USA; LSM710).

### Measurement of HLMVEC permeability (PA) by transendothelial resistance (TER) and fluorescein isothiocyanate (FITC)

A monolayer of HLMVECs was seeded on 24-well Transwells (Corning Inc., Corning, CA, USA) with 0.4-μm pore membranes. The apical chamber of the Transwell was hydrated overnight with medium, HLMVECs (2 × 10^5^ cells/mL) were suspended in serum-free medium and added to the apical chamber, and 600 μL of cell serum medium was added to the basolateral chamber. Cells were incubated and observed with an inverted microscope until steady state was reached to establish an in vitro monolayer cell model in Transwells. After the addition of different treatments, two electrodes (EVOM; World Precision Instruments, Sarasota, FL, USA) were used to measure the electrical impedance value across the endothelial cells or the cell-free membrane insert, with one electrode placed in the apical chamber and the other in the basolateral chamber. The electrical impedance value across the endothelial cell monolayer was determined using identical testing configuration settings as those used to measure the cell-free membrane. The cell-specific resistance was calculated by subtracting the resistance of the cell-free membrane from the resistance of the measured cell monolayer. Then the TER was determined by multiplying the bottom area of the Transwell cell (0.33 cm^2^) (unit: Ω·cm^2^); the permeability coefficient across HLMVECs was determined by measuring the ratio of the TER value to the corresponding resting state TER value.

Next, 200 μL of FITC–dextran solution (500 μg/mL), which had been prepared with incomplete EBM-2 medium, was placed the apical chamber of the Transwell, followed by the addition of 600 μL in EBM-2 incomplete medium in the lower chamber. The chamber was subsequently incubated at 37 °C with 5% CO_2_ for 1 h, and 100 μL was transferred from the apical or basolateral chamber into a black 96-well plate. The fluorescence intensities of the samples from both the apical and basolateral chambers were measured using a Synergy II multifunction enzyme labeling instrument (BioTek Company, Winooski, VT, USA).

An excitation wavelength of 495 nm and an emission wavelength of 520 nm were used. The permeability of the endothelial monolayer to dextran is expressed as the permeability coefficient PA, and the PA was calculated with the following formula: PA = [*A*]/*T* × 1/*A* × *V*/[*L*].

Note: [*A*] is the fluorescence intensity value of the lower chamber; *T* indicates the incubation time of FITC dextran (unit: s); *A* is the area of the filter membrane of the small chamber (unit: cm^2^); *V* is the liquid volume in the basolateral chamber; and [*L*] is the fluorescence intensity value of the apical chamber. The experimental results are expressed as PA%. PA% = (experimental sample group PA/experimental control group PA) × 100%.

### Dual luciferase reporter gene assay

The dual luciferase reporter experiment was performed using HEK293 cells (CRL1573, ATCC) that were cultured in 48-well plates for 24 h. Next, the psicheck2 luciferase reporter plasmid (Promega, Madison, WI, USA) was used to construct ATG7 3’-untranslated region wild-type (WT, 5’-CCUGUACAUUCUUUACGCACAG-3’) or mutant (MUT, 5’-AGUCGGCGACAGUGACTACAAG) plasmids, which were cotransfected with 50 nmol/L miR-210-3p mimic or negative control (mimic-NC) into HEK293 cells for a period of 48 h. The dual fluorescence luciferase reporter gene system (Promega) was used to examine the fluorescence of Renilla luciferase (Rluc) and Firefly luciferase (Luc) on a microplate reader (Lumat LB 9508, EG&G Berthold, Wildbad, Germany). The relative luciferase activity was determined based on the ratio of Luc to Rluc, with Rluc as the internal reference, which allowed verification of whether ATG7 was a direct target gene of miR-210-3p. The experiment was repeated three times in an independent manner.

### Flow cytometry

An Annexin V-FITC/Propidium Iodide Staining Kit (BD Biosciences, San Diego, CA, USA) was used to analyze apoptosis. Briefly, BEAS-2B cells were plated in a six-well plate. Upon reaching 70% confluence, the cells were treated with Con-EVs or septic-EVs for 24 h. The cell supernatant was then collected, after which the cells in the culture supernatant were centrifuged. The cells were trypsinized in the absence of ethylenediaminetetraacetic acid (EDTA), centrifuged, and washed twice with PBS, together with the cells in the supernatant. Annexin V-FITC/propidium iodide was added to the cells and incubated for 15 min according to the manufacturer’s instructions. All cell samples were then sorted and subsequently analyzed using a flow cytometer (BD FACSVerse) at an excitation wavelength of 488 nm and an emission wavelength of 525 nm.

### Endothelial tubule formation

Matrigel (356234, Corning) was stored at −20 °C and thawed gradually at 4 °C prior to the experiment. Next, 5 μL of ice-cold Matrigel solution was placed into a 96-well plate and air dried. HLMVECs were harvested using trypsin-EDTA solution, after which they were seeded on Matrigel-coated 96-well plates at a density of 3 × 10^4^ cells/mL and cultured with serum-free medium. The cells were incubated with 20 μM calcein-AM (Sigma Aldrich) for 15 min, and cell morphology and tubule formation were analyzed under a fluorescence microscope (Nikon, Tokyo, Japan). All data were analyzed using the ImageJ software.

### Quantitative reverse transcription polymerase chain reaction (qRT-PCR)

Total RNA was extracted using a TRIzol Kit (15596026, Thermo Fisher). Synthesis of complementary DNA (cDNA) from mRNA was performed using a commercially available kit (Cat# RR037A, Takara, Tokyo, Japan) according to the instructions provided by the manufacturer. Synthesis of miR-144 cDNA was performed using the miRNA First Strand cDNA Synthesis Kit (Tailing Reaction) (B532451-0020) based on the manufacturer’s instructions (Sangon Biotech, Shanghai, China). Next, the cDNA was subjected to real-time quantitative PCR using a SYBR^®^ Premix Ex TaqTM II (Perfect Real Time) Kit (DRR081, Takara) with an ABI 7500 instrument (ABI, USA), and each reaction was run in triplicate. Universal RT primers for miRNAs and forward primers for U6 were provided by the miRNA First Strand cDNA Synthesis Kit. The primers used are listed in Table S1. GAPDH or β-actin was used as the internal reference for both cell and tissue mRNA analysis, U6 was used as the internal reference for cell miR-210–3p analysis, and cel-miR-39 served as the external reference for plasma and plasma EV miR-210-3p analysis. The relative expression level of mRNA or miRNA was calculated using the 2^−ΔΔCt^ method: ΔΔCt = (average Ct value of the target gene of the experimental group − average Ct value of the housekeeping gene of the experimental group) − (average Ct value of the target gene of the control group − control group housekeeping gene average Ct value). The Ct represents the number of amplification cycles that were required to pass when the real-time fluorescence intensity of the reaction reached the set threshold during logarithmic amplification. The experiment was repeated three times in an independent manner.

### Bioinformatics analysis

The TargetScan (http://www.targetscan.org/vert_71/), starBase (http://starbase.sysu.edu.cn/index.php), and mirDIP (http://ophid.utoronto.ca/mirDIP/index.jsp#r) databases were examined to predict the downstream target genes of miR-210-3p. The gene expression dataset GSE69345, which is related to cecal ligation and puncture (CLP) murine models, in the GEO database was downloaded and included 12 CLP mouse sepsis model samples and 4 control samples. R language “limma” package differential analysis was performed, with |logFC| > 0.34 and *P* < 0.05 set as the screening criteria for differentially expressed genes. Finally, the database prediction results intersected with the mRNAs in the GSE69345 dataset that were significantly downregulated.

### Establishment of the mouse sepsis model

All mice were housed individually in specific pathogen-free-grade animal laboratory cages and provided free access to food and water. The mice were maintained under a 12-h light/dark cycle at room temperature (22–25 °C) with 60–65% humidity. The experiment was initiated following 1 week of adaptive feeding, and the health status of the mice was evaluated regularly prior to the start of the experiment. The mice were subsequently fasted for 12 h before surgery and randomly divided into three groups: the sham operation group (sham), the CLP + Ad-NC group, and the CLP + Ad-anti-miR-210-3p group. Three days prior to the operation, 50 μL of adenovirus with a titer of 10^12^ pfu/mL was injected through the tail vein into the mice using a 30-G insulin needle. CLP was used to establish the sepsis mouse model. Following intraperitoneal anesthesia with an injection of 2% pentobarbital sodium, the mice were fixed in the supine position on the experimental table, and a 1.5-cm incision was made at the midline of the abdomen. Sterile toothless forceps were used to free the mesentery and cecum. The cecum was ligated circularly using sterile No. 3 silk thread at the appropriate position and perforated through the blind end with a 21-gauge needle. After extruding the intestinal contents, the abdominal cavity was opened, after which the abdominal cavity was sutured layer by layer. After the operation, 1 mL of saline was injected subcutaneously into the back region for fluid resuscitation purposes. The Sham group underwent an operation using the same method, with the exception of cecal ligation and perforation, which were not performed.

### Observation of the 7-day survival rates of septic mice

We observed the survival of the mice every 24 h after the CLP operation, and the daily survival rates of the mice in each group were recorded. Next, 18 mice were randomly selected from each group, and their feeding, changes in activity, and survival were evaluated every 4 h to establish a survival curve.

### Measurement of inflammatory factors in mouse plasma and bronchoalveolar lavage fluid (BALF) by enzyme-linked immunosorbent assay (ELISA)

The levels of the inflammatory factors IL-1β (1210122, Mouse IL-1β ELISA Kit 96T, Dakewe, Shenzhen, China), tumor necrosis factor-α (TNF-α, 1217202, Mouse TNF-α ELISA Kit 96T, Dakewe), and IL-6 (1210602, Mouse IL-6 ELISA Kit 96T, Dakewe) were measured. As per the instructions, 100 μL of cells were incubated with the biotinylated antibody working solution (1:100, 100 μL/well) for 2 h, and the optical density value was measured at 450 nm and calculated by comparison to the standard and blank. The experiment was repeated three times in an independent manner.

### Pulmonary wet-to-dry weight (*W*/*D*) ratio

The upper right lungs were removed from the mice in each group, after which the water and blood on the surface were blotted using absorbent paper. The lungs were weighed using an electronic scale. The lungs were placed in a 60 °C oven for 72 h until reaching a constant weight and were then weighed. These data were recorded as the dry weight, after which the lung *W*/*D* ratio was calculated.

### Histopathology

Middle lobe lung tissues were fixed with 10% neutral paraformaldehyde, routinely dehydrated, embedded in paraffin, dewaxed with xylene, dehydrated with gradient ethanol, and sliced at a thickness of 5 μm. Next, hematoxylin–eosin (HE) staining was performed on three randomly selected slices from each mouse. The severity of lung tissue damage was examined and evaluated using a microscope.

### Pulmonary microvascular leakage test

Evans blue staining was performed by injecting Evans blue (0.5 mL, 50 μg/g) into the tail vein 30 min prior to sampling, after which the pulmonary circulation was rinsed with 10 mL of PBS. After the lung tissues had been excised, the tissues were promptly rinsed with PBS and frozen in liquid nitrogen. The lung tissues were homogenized in ice-cold PBS, after which the homogenate was incubated with formamide at 60 °C for 16 h. After being centrifuged at 7000 × *g* for 25 min, the supernatant was collected and placed into a multifunctional microplate reader (BioTek), and the absorbance (A620) was measured to determine the Evans blue levels in the tissues.

### Immunohistochemical analysis of myeloperoxidase (MPO) and von Willebrand factor (vWF)

The tissue sections were subjected to antigen retrieval and washed three times with PBS (5 min each time). A circle was drawn around the slices using a crayon, after which 3% hydrogen peroxide solution was added to the slices and incubated at room temperature for 15 min, followed by antigen repair. The dewaxed and hydrated tissue sections were placed into sodium citrate–citric acid antigen repair buffer, boiled at high pressure for 10 min in an autoclave, subjected to antigen repair, and cooled naturally to room temperature. Antigen blockade was then performed, after which normal goat blocking serum was added to the sections and incubated at room temperature for 15 min. After the blocking serum had been removed, diluted primary antibody solution (MPO, 1:25, ab9535, Abcam; vWF 1:50, IR527, FLEX, Dako, Glostrup, Denmark) was added to the sections and incubated in a humidified chamber at 4 °C overnight. The following day, the slices were removed, diluted fluorescent secondary antibody (1:100, Abcam) was added, and the slices were incubated at room temperature for 30 min. The cell nuclei were stained with DAPI and photographed under a microscope.

### Terminal deoxynucleotidyl transferase dUTP nick end labeling (TUNEL) staining

Apoptosis in lung tissue was examined using the Dead End Fluorometric TUNEL System Kit (G3250; Promega), which was used to stain the paraffin-embedded lung tissue sections. The TUNEL-stained sections were photographed under a microscope, and the numbers of apoptotic cells in each field of view were calculated.

### Statistical analysis

SPSS version 21.0 (IBM Corp. Armonk, NY, USA) was used for statistical analyses. Measurement data are expressed as mean ± standard deviation. Unpaired *t* tests were used to compare data between two groups. One-way analysis of variance (ANOVA) followed by Tukey’s posttest was used to compare data among multiple groups, and the data at different time points among multiple groups were compared using Bonferroni-corrected repeated-measures ANOVA. Kaplan–Meier curves were used for survival analysis, and differences were determined using log-rank test. A value of *P* < 0.05 indicates that the difference was statistically significant.

## Results

### Clinical index

Thirty healthy individuals were recruited as control subjects, in addition to 55 septic patients. As shown in Table S2, there was no significant difference in terms of sex, age, or body mass index between the control subjects and septic patients (*P* > 0.05), while the APACHE II and SOFA scores of septic patients were all significantly higher than those of the control subjects (*P* < 0.05). Moreover, an increased risk of ALI was observed in septic patients, and there were 15 cases of pneumonia, 3 cases of blood transfusions, and 2 cases of pancreatitis.

### Increased expression of miR-210-3p in plasma EVs during sepsis

**Fig. 1:**
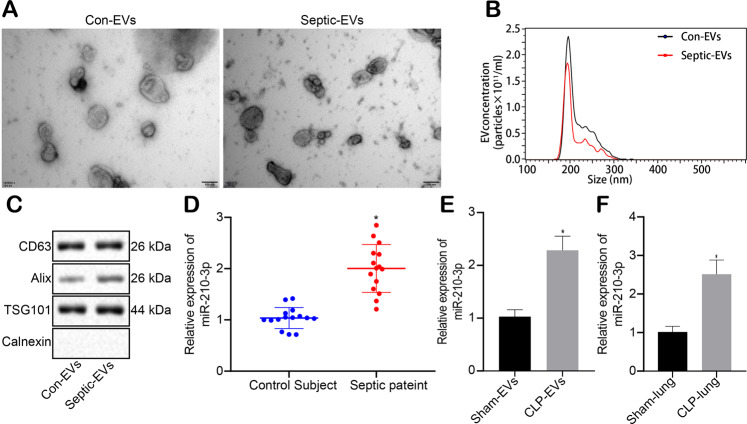
Increased expression of miR-210-3p in plasma EVs in sepsis. Electron microscopic analysis of EV morphology and diameter (scale bar: 100 nm) (**a**). NTA of EV diameter and number (**b**). Western blot analysis of the expression of EV surface marker proteins CD63, Alix, and TSG101 (**c**). Measurement of human plasma EV miR-210-3p expression by qRT-PCR (**d**). Measurement of mouse plasma EV miR-210-3p expression by qRT-PCR (**e**). Measurement of mouse lung tissue miR-210-3p expression by qRT-PCR (**f**). *P < 0.05. The values are all measured data that are expressed as mean ± standard deviation, and unpaired *t* tests were used between the two groups.

The expression of microRNAs such as miR-210-3p, miR-145, and miR-122 was significantly elevated in CLP-induced animal blood and cells^15,16^. We collected peripheral blood from both healthy subjects and septic patients to obtain plasma EVs. The EVs were identified as sphere-shaped vesicles using TEM (Fig. 1a). Con-EVs and septic-EVs were similar in structure and size, and their average diameter was assessed using NTA at a range of 100–200 nm (Fig. 1b). Western blot analysis revealed that the EVs expressed surface marker proteins (ALIX, CD63, and TSG101) (Fig. 1c), indicating that the protein levels of the septic patients were significantly higher than those of the healthy subjects. Additionally, qRT-PCR analysis revealed that the expression of miR-210-3p was elevated in septic-EVs relative to Con-EVs (Fig. 1d). We also collected mouse plasma EVs to measure the expression of miR-210-3p, and the results were similar to those in humans (Fig. 1e). The expression of miR-210-3p was upregulated in the lung tissues of septic mice (Fig. 1f). In summary, the expression of miR-210-3p is increased in plasma EVs in sepsis.

### Sepsis plasma EVs promote inflammation and apoptosis

**Fig. 2:**
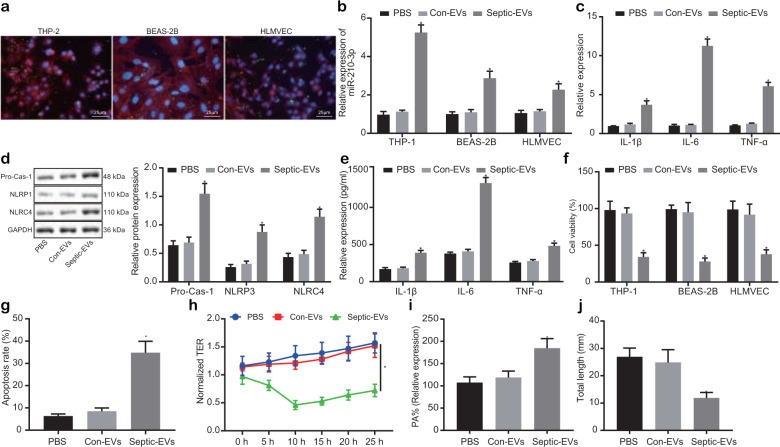
Sepsis plasma EVs promote inflammation and apoptosis. Laser confocal observation of huc-MSC-EVs labeled with fluorescent PKH67 uptake by THP-1 cells, BEAS-2B cells, and HLMVECs (×400) (**a**). PKH67-labeled EVs are green, and DAPI-stained nuclei are blue. The phalloidin-labeled cytoskeleton is stained red; scale: 20 μm. Measurement of miR-210-3p expression in the three cell lines by qRT-PCR (**b**). Measurement of THP-1 macrophage expression of the inflammatory markers IL-1β, IL-6, and TNF-α by qRT-PCR (**c**). Western blot measurement of inflammatory body expression markers (**d**). ELISA measurement of THP-1 cell supernatant expression of inflammatory factors (**e**). CCK-8 detection of THP-1 cell viability (**f**). Flow cytometry was used to measure apoptosis in BEAS-2B cells (**g**). Transendothelial resistance was used to measure endothelial cell TER (**h**). FITC fluorescence analysis of endothelial cells PA% (**i**). Matrigel analysis of endothelial cell tubule formation (j). *P < 0.05 vs Con-EVs. These values are all measured data that are expressed as mean ± standard deviation. One-way ANOVA and Tukey’s test were used for post hoc comparisons between multiple groups. Data were compared between groups at different time points, repeated-measures analysis of variance was used, and Bonferroni’s test was used for post hoc analysis. The experiment was repeated three times.

Next, to ascertain the effect and mechanism of EVs derived from septic patients on lung tissue damage, three types of cells related to lung tissue (macrophages (THP-1 cells), BEAS-2B cells, and HLMVECs) were cultured with 20 μg of plasma EVs. After 12 h of cell culture with PKH-67-labeled EVs, we identified abundant plasma EVs in the cells, which were distributed around the nucleus (Fig. 2a). Furthermore, EVs from healthy controls and septic patients were incubated with the three kinds of cells for 24 h, and qRT-PCR was performed to measure the expression of miR-210-3p, and the results demonstrated that, compared with that in the Con-EV group, the expression of miR-210-3p in the septic-EV group increased (Fig. 2b), and THP-1 macrophages were significantly increased. Next, qRT-PCR and ELISA were used to measure the expression of macrophage inflammatory factors (IL-1β, IL-6, and TNF-α), and the results revealed the overproduction of inflammatory factors, with excessive levels of IL-6 in the septic-EV group (Fig. 2c). Furthermore, we measured the expression of inflammation-related pathways and inflammatory bodies by western blotting (Fig. 2d), and the results indicated the upregulation of pro-caspase1, NLRP3, and NLRC4 expression in the septic-EV group, indicating that septic-EVs induced the development of inflammation in septic patients. Moreover, the results of ELISA in the supernatant of THP-1 macrophages were similar to the results of qRT-PCR (Fig. 2e). Furthermore, our results highlighted the limited amount of THP-1 cell proliferation (Fig. 2f) and increased apoptosis in BEAS-2B lung epithelial cells in the presence of septic-EVs (Fig. 2g). For pulmonary microvascular endothelial cells, we used TER measurement and FITC to examine permeability. Our results illustrated that the TER value of endothelial cells decreased after treatment with septic-EVs, with the lowest TER was detected at 10 h (Fig. 2h). An inverse relationship was identified regarding the PA% value, which was increased following septic-EV challenge (Fig. 2i), indicating that septic-EVs reduced endothelial cell barrier function while increasing permeability. We observed impaired tubule formation in HLMVECs after septic-EV insult (Fig. 2j), while no such observation was made in the control group. Therefore, we concluded that septic-EVs increase inflammation, apoptosis, and endothelial cell permeability, resulting in ALI.

### MiR-210-3p promotes inflammation and apoptosis

**Fig. 3:**
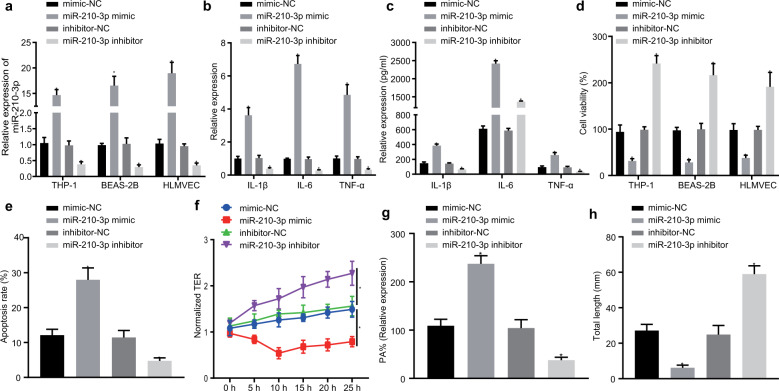
MiR-210-3p promotes inflammation and apoptosis. Measurement of miR-210-3p expression by qRT-PCR (**a**). Measurement of THP-1 macrophage inflammatory factor expression by qRT-PCR (**b**). ELISA analysis of inflammatory factor expression in the cell supernatant (**c**). CCK-8 analysis of cell viability (**d**). Flow cytometry was used to examine apoptosis (**e**). Transendothelial resistance was used to measure endothelial cell TER (**f**). FITC fluorescence analysis of endothelial cell PA% (**g**). Matrigel analysis of endothelial cell tubule formation (**h**). *P < 0.05 vs mimic-NC or inhibitor-NC; the values are all measurement data that are expressed as mean ± standard deviation. Unpaired *t* tests were used for comparisons between the two groups, and for comparisons between the groups at different time points, repeated-measures analysis of variance and the Bonferroni posttest were used. The experiment was repeated three times.

Previous studies^15,16^ and prediction software have suggested that miR-210-3p is highly expressed in the serum and plasma of patients with sepsis. However, the mechanism of miR-210-3p in ALI remains unclear. Hence, we transfected three cell lines with mimic-NC, inhibitor-NC, the miR-210-3p mimic, and the miR-210-3p inhibitor and measured the expression of miR-210-3p by qRT-PCR (Fig. 3a). Our results suggested that the miR-210-3p mimic could promote the secretion of inflammatory factors by fourfold or greater, while the miR-210-3p inhibitor suppressed these factors by half (Fig. 3b, c). CCK-8 was used to examine cell viability after different transfection conditions, and the results indicated that, compared with mimic-NC, the miR-210-3p mimic markedly reduced cell viability and that the miR-210-3p inhibitor increased cell viability (Fig. 3d). BEAS-2B cell apoptosis was evaluated, and the results suggested that the miR-210-3p mimic increased BEAS-2B cell apoptosis, while the miR-210-3p inhibitor inhibited BEAS-2B cell apoptosis (Fig. 3e). TER and cell permeability were analyzed, and the miR-210-3p mimic inhibited the TER value of pulmonary microvascular endothelial cells, particularly at the 10-h time point (Fig. 3f). The permeability coefficient PA% increased significantly, while the miR-210-3p inhibitor induced the opposite trend (Fig. 3g). Finally, tubule formation was analyzed in transfected lung microvascular endothelial cells, and we found (Fig. 3h) that the miR-210-3p mimic significantly inhibited pulmonary microvascular endothelial tubule formation compared to mimic-NC, while the opposite effect was observed following miR-210-3p inhibitor treatment. In summary, miR-210-3p can promote the development of inflammation and apoptosis, which may further contribute to ALI.

### Bioinformatics analysis identified ATG7 as a major downstream target gene of miR-210-3p

**Fig. 4:**
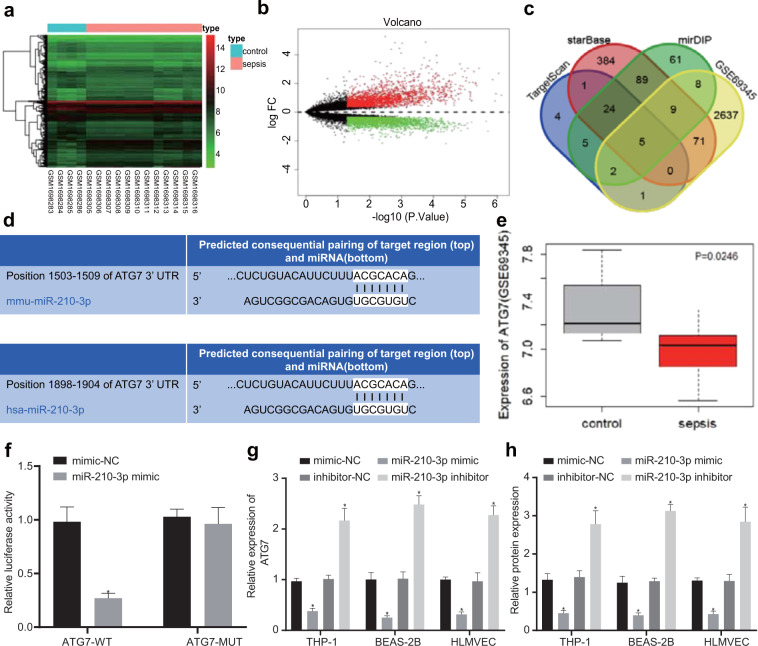
Prediction of downstream regulatory genes of miR-210-3p in the context of sepsis. GSE69345 chip screening of CLP mouse sepsis model samples and heat map showing differential gene expression. The abscissa represents the sample number, the ordinate represents the gene name, and each small square in the figure represents the expression of a gene at the sample level (**a**). GSE69345 chip screening of CLP mouse sepsis model samples showing differential gene expression in a volcano map; the black part indicates no significant difference in mRNA, green represents CLP mouse sepsis model sample mRNA expression that was significantly downregulated, and red represents significantly upregulated mRNA in CLP mouse sepsis model samples (**b**). MiR-210-3p downstream regulatory gene prediction; blue circles represent target genes predicted by the TargetScan database, red circles represent targets predicted by the StarBase database, green circles indicate target genes predicted by the mirDIP database, and yellow circles indicate genes that were significantly downregulated in the GSE69345 chip of septic mouse samples (**c**). ATG7- and miR-210-3p-binding site map (**d**). ATG7 expression in the SSE mouse chip GSE69345 (**e**). Dual luciferase reporter gene experiment; *P < 0.05 vs mimic-NC + ATG7-WT (**f**). qRT-PCR analysis of the mRNA expression of ATG7 after expressing miR-210-3p and inhibiting miR-210-3p (**g**). Western blot analysis of the protein expression of ATG7 after overexpressing miR-210-3p (**h**).

In an attempt to further elucidate the mechanism of miR-210-3p in patients with sepsis, downstream target genes of miR-210-3p were predicted through the TargetScan, StarBase, and mirDIP databases. We identified 42, 583, and 204 target genes, respectively. Furthermore, we screened the CLP mouse sepsis model-related mRNA expression chip GSE69345 from the GEO database, with a |logFC| > 0.34 and *P* < 0.05 as the screening standard. The R language “limma” package was used for differential analysis. In samples from the sepsis model, 2298 significantly upregulated and 2733 significantly downregulated mRNAs were identified (Fig. 4a, b). Next, the prediction results from various databases, including TargetScan, were compared with mRNAs that were significantly downregulated in the GSE69345 chip of septic mice (Fig. 4c, d). The results indicated that five mRNAs (CLUH, ATG7, KMT2D, PPTC7, and NFIX) were present in these four datasets, among which ATG7 was the most significantly underexpressed in septic mice in dataset GSE69345 (Fig. 4e). The dual luciferase reporter experiment revealed that the miR-210-3p mimic significantly suppressed the luciferase activity of ATG7-WT, while no such effect on the luciferase activity of ATG7-MUT was detected. Compared with that in the mimic NC group, the luciferase signal of ATG7-WT in the miR-210-3p mimic group was decreased (*P* < 0.05), which indicated that miR-210-3p could bind specifically to ATG7 (Fig. 4f). qRT-PCR and western blot analysis demonstrated that the mRNA and protein expression of ATG7, respectively, was decreased by the miR-210-3p mimic, but the opposite effect was observed in response to the miR-210-3p inhibitor (Fig. 4g, h). In summary, the delivery of miR-210-3p by peripheral circulating EVs may play a role in ALI in the context of sepsis by targeting ATG7.

### MiR-210-3p targeting of ATG7 inhibits autophagy

**Fig. 5:**
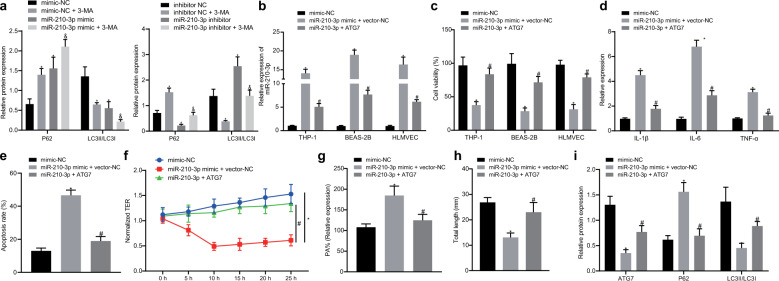
MiR-210-3p targeting of ATG7 inhibits the expression of autophagy-related genes. Western blot analysis of LC3II, LC3I, and P62 protein expression levels after the overexpression or inhibition of miR-210-3p or 3-MA treatment (**a**). Measurement of miR-210-3p expression by qRT-PCR (**b**). CCK-8 analysis of cell viability (**c**). Measurement of THP-1 cell inflammatory factors by qRT-PCR (**d**). Flow cytometric analysis of early and late apoptosis in BEAS-2B cells (**e**). Transendothelial resistance in endothelial cells was determined by the TER method (**f**). FITC fluorescence was used to examine endothelial cell permeability (**g**). Matrigel was used to measure endothelial cell angiogenesis (**h**). Western blot analysis of ATG7 and P62 expression and the LC3II/LC3I ratio (**i**). *P < 0.05 vs mimic-NC, ^&^*P* < 0.05 vs miR-210-3p mimic-NC or miR-210-3p inhibitor, ^#^*P* < 0.05 vs miR-210-3p+vector-NC; the values are all measured data and are expressed as mean ± standard deviation. One-way ANOVA and Tukey’s test were used for post hoc comparisons between multiple groups. The data were compared between groups at different time points, repeated-measures analysis of variance was used, and Bonferroni’s test was used for post hoc inspection. The experiment was repeated three times.

The loss of the autophagy-related protein ATG7 could induce IL-1β expression and activate the NLRC4 inflammatory body^17^. High levels of IL-1β secretion have been well documented in the activation of inflammation in the body^18^. ATG7 deletion may also inhibit autophagosome formation. The biomarker of autophagy is the formation of LC3II. LC3II is converted from LC3I in autophagosomes^19^. ATG7 deficiency has been reported to contribute to the accumulation of P62, which is known as an autophagy substrate protein^20^. Sepsis has been widely suggested to be an inducer of ALI. MiR-210-3p regulates the expression of autophagy proteins by targeting ATG7. We performed western blot analysis and found suppressed levels of P62 and enhanced LC3II/LC3I ratios following the overexpression of miR-210-3p, while the fluctuations in these indicators suggested autophagy dysfunction. The results also indicated a marked elevation in the expression of P62 and a reduction in the LC3II/LC3I ratio after the addition of the autography inhibitor 3-MA, whereas this autophagy dysfunction was reversed by miR-210-3p inhibitor treatment after 3-MA challenge (Fig. 5a). Next, to further ascertain the protective mechanism of ATG7 in lung injury, we overexpressed ATG7 and transfected cells with miR-210-3p. MiR-210-3p expression was dramatically upregulated in the miR-210-3p mimic + vector-NC group but was downregulated due to the presence of ATG7 (Fig. 5b). Moreover, the miR-210-3p mimic + ATG7 group exhibited notably higher cell viability and less inflammatory factor release than the miR-210-3p + vector-NC group (Fig. 5c, d). The role of ATG7 in BEAS-2B cells was also investigated using flow cytometry in an attempt to verify the protective effects of ATG7 on apoptosis (Fig. 5e). ATG7 protected endothelial barrier function after challenge with miR-210-3p, as indicated by an increased TER value (Fig. 5f) and reduced permeability (Fig. 5g). The ability of endothelial cells to form tubules was further examined, and the results are shown in Fig. 5h. Compared with that in the miR-210-3p mimic + vector NC group, endothelial neovascularization was increased in the miR-210-3p mimic + ATG7 group. Stimulated endothelial angiogenesis was indicated by increases in the expression of ATG7 and the LC3II/LC3I ratio and a decrease in P62 (Fig. 5i). Taken together, these results suggest that miR-210-3p inhibits ATG7 expression and autophagy.

### Inhibiting miR-210-3p expression in septic EVs attenuated inflammation and apoptosis

**Fig. 6:**
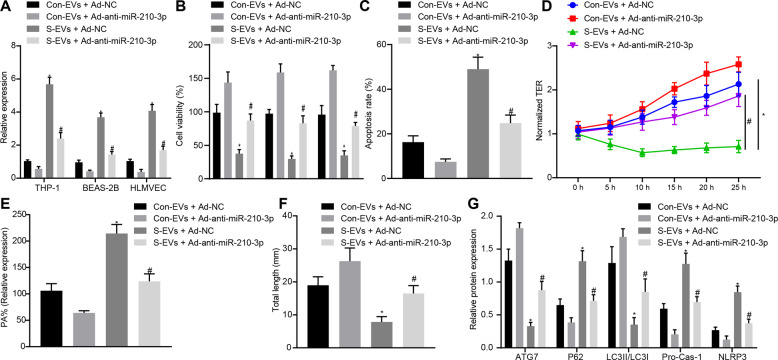
Inhibition of miR-210-3p expression in septic EVs relieves inflammation and apoptosis. Measurement miR-210-3p expression by qRT-PCR (**a**). CCK-8 analysis of cell viability (**b**). Flow cytometric analysis of BEAS-2B cell apoptosis (**c**). Transendothelial resistance analysis of endothelial cell TER (**d**). FITC fluorescence analysis of the endothelial cell permeability coefficient PA (**e**). Matrigel analysis endothelial cell angiogenesis (**f**). Western blot analysis of protein expression in each treatment group (**g**). *P < 0.05 vs Con-EV + Ad-NC; ^#^*P* < 0.05 vs S-EV + Ad-NC. The values are all measured data that are expressed as mean ± standard deviation. One-way ANOVA and Tukey’s test were used for post hoc comparisons between multiple groups. The data were compared between groups at different time points, repeated-measures analysis of variance was used, and Bonferroni’s test was used for post hoc inspection. The experiment was repeated three times.

After THP-1 cell, BEAS-2B cell, or HLMVEC uptake of either Con-EVs or septic-EVs for 24 h, the adenovirus Ad-anti-miR-210-3p, which is a miR-210-3p inhibitor, was administered and significantly downregulated miR-210-3p expression in both the Con-EV and septic-EV groups compared to the negative adenovirus control group (Fig. 6a). Although septic-EVs attenuated cell viability, Ad-anti-miR-210-3p improved cellular conditions, as indicated by increased cell growth (Fig. 6b). Flow cytometry indicated that BEAS-2B cell apoptosis was promoted in the S-EV + Ad-NC group, while apoptosis was reduced in the S-EV + Ad-anti-mIR-210-3p group (Fig. 6c). We subsequently evaluated the permeability of pulmonary microvascular endothelial cells and found that S-EV + Ad-NC reduced the TER value (Fig. 6d), while the S-EV + Ad-anti-mIR-210-3p group had a significantly increased TER value. Compared to that in the Con-EV + Ad-NC group, the PA% of the S-EV + Ad-NC group was significantly increased, while the PA% values of the S-EV + Ad-anti-miR-210-3p group were decreased compared to those of the S-EV + Ad-NC group (Fig. 6e). The tubule formation results were similar to the PA% results (Fig. 6f). The protein levels of ATG7, P62, NLRP3, and IL-1β and the LC3II/LC3I ratio are shown in Fig. 6g. The S-EV + Ad-NC group exhibited improved expression of the inflammatory markers IL-1β and NLRP3 and inhibited protein expression of ATG7. The ratio of LC3II/LC3I decreased and the expression of P62 increased, while the S-EV + Ad-anti-miR-210-3p group exhibited the opposite effects. In summary, sepsis-associated plasma EVs may induce ALI through targeted inhibition of ATG7 and autophagy by miR-210-3p. Inhibiting miR-210-3p expression may alleviate ALI.

### Mouse plasma EVs may induce ALI by inhibiting ATG7 through miR-210-3p

**Fig. 7:**
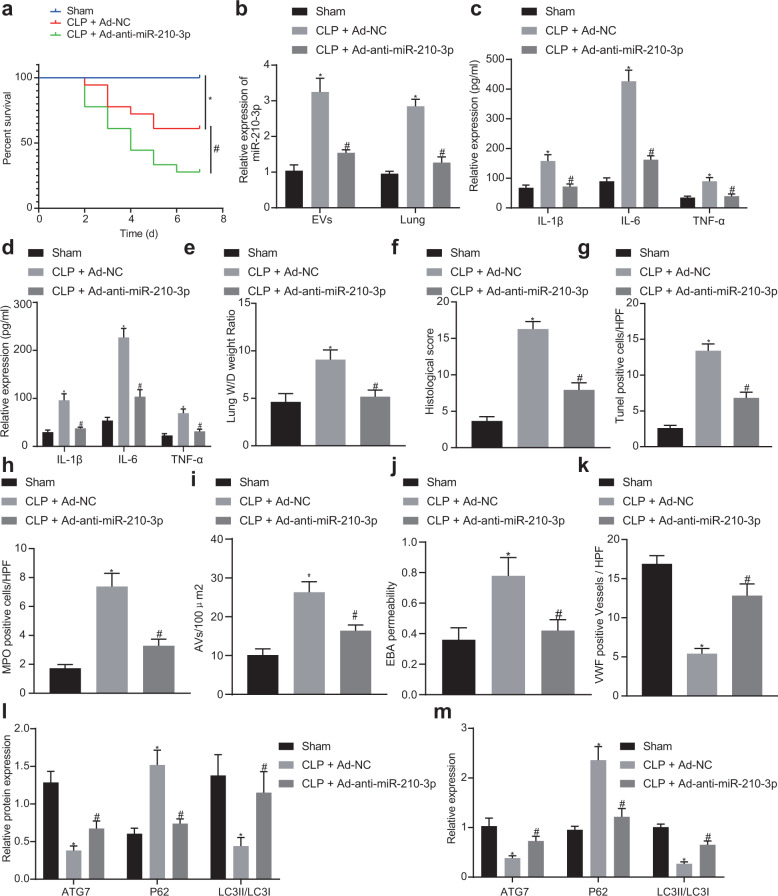
Mouse plasma EVs may inhibit ATG7 expression through miR-210-3p targeting and induce ALI. KM survival curve analysis of the 7-day survival rates of the three groups of mice (**a**). Analysis of miR-210-3p expression in mouse plasma EVs and lung tissue by qRT-PCR (**b**). ELISA analysis of the expression of the inflammatory factors IL-1β, IL-6, and TNF-α in the plasma of Hefei alveolar lavage fluid (**c**, **d**). Weighing method to analyze the wet/dry weight ratio of lung tissue (**e**). HE staining to determine pathological changes in lung tissue structure (**f**). TUNEL staining to examine lung tissue apoptosis (**g**). MPO staining to examine lung tissue neutrophil infiltration (**h**). Evans blue dye analysis of lung tissue permeability (**i**). Immunohistochemical analysis of vWF-positive blood vessels in lung tissue (**j**). Western blot analysis of ATG7 and autophagy protein expression in lung tissue (**k**). Analysis of lung tissue ATG7 and autophagy-related gene mRNA expression by qRT-PCR (**l**). Electron microscopic ultrastructural analysis of small autophagic bodies in lung tissue (**m**). *P < 0.05 vs sham, ^#^*P* < 0.05 vs CLP + Ad-NC. The values are all measured data and are expressed as mean ± standard deviation. One-way ANOVA and Tukey’s post hoc test were used for data comparisons between multiple groups. The data were compared between groups at different time points, repeated-measures analysis of variance was used, and Bonferroni’s test was used for post hoc inspection. The experiment was repeated three times.

Tail vein injection of Ad-NC or Ad-anti-miR-210-3p was performed, and the CLP surgery was performed 2 days later. Some mice were sampled after 24 h, while the remaining mice were observed over a 7-day period. Compared with that of the sham group, the 7-day survival analysis (Fig. 7a) revealed that the mortality rate of mice in the CLP + Ad-NC group was significantly increased, while that of mice in the CLP + Ad-anti-miR-210-3p group was reduced. The plasma and lung tissues of mice were collected to measure the expression of miR-210-3p in plasma EVs and lung tissue (Fig. 7b). Compared with that in the sham group, the expression of miR-210-3p in the CLP + Ad-NC group was increased, while the expression in the CLP + Ad-anti-miR-210-3p group was decreased. The expression of inflammatory factors in plasma and alveolar lavage fluid was measured by ELISA (Fig. 7c–d). The CLP + Ad-anti-miR-210-3p group exhibited decreased expression of inflammatory factors. The *W*/*D* ratio of the lung was calculated after CLP injury, the result of which indicated that the ratio in the Ad-NC group was increased, while the ratio was reduced in the Ad-anti-miR-210-3p group, suggesting less severe pulmonary edema accompanied by reduced accumulation of fluid in the Ad-anti-miR-210-3p group (Fig. 7e). HE staining of mouse lung tissues in each group (Fig. 7f) revealed that the alveolar structure of mice in the sham group was intact. The lung tissues in the CLP + Ad-NC group had widened alveolar septa and infiltration of a large number of inflammatory cells into the alveolar wall and interstitium.

The TUNEL staining (Fig. 7g) results indicated that the number of TUNEL-positive cells in the CLP + Ad-NC group was significantly increased. The number of TUNEL-positive cells in the lung tissue of the CLP + Ad-anti-miR-210-3p group decreased significantly (*P* < 0.05). In addition, MPO staining revealed that the number of MPO-positive neutrophils in the CLP + Ad-NC group increased significantly, whereas that in the CLP + Ad-anti-miR-210-3p group decreased (Fig. 7h). Evans blue dye was subsequently used to detect leakage in lung tissue (Fig. 7i). The Evans blue albumin permeability in the CLP + Ad-NC group was significantly higher than that in the sham group, whereas that in the CLP + Ad-anti-miR-210-3p group was decreased. Immunohistochemical analysis of vWF staining in lung tissue sections demonstrated that blood vessel density was significantly increased in the CLP + Ad-anti-miR-210-3p group (Fig. 7j). Finally, we measured the expression of ATG7 and autophagy-related proteins in lung tissues of the three groups by western blotting. The results suggested that the protein expression of ATG7 protein and the LC3II/LC3I ratio were inhibited in the CLP + Ad-NC group, and the production of P62 was promoted (Fig. 7k). The RNA and protein expression results were similar the previous results (Fig. 7l). TEM was used to detect autophagosomes in lung tissue. The results indicated that autophagosomes in the CLP + Ad-NC group were significantly increased compared with those in the sham group. Compared with those in the CLP + Ad-NC group, autophagosomes were reduced in the CLP + Ad-anti-miR-210-3p group (Fig. 7m). Taken together, these results suggest that miR-210-3p carried by plasma EVs was increased in CLP-induced sepsis mice and stimulated the lung tissue secretion of inflammatory factors via ATG7 to induce ALI.

## Discussion

In the present study, we aimed to elucidate the role of miR-210-3p in regulating autophagy and inflammatory activation in septic ALI and the intrinsic mechanism, and our results indicated that plasma EV-mediated delivery of miR-210-3p targets ATG7 to regulate autophagy and inflammatory activation in sepsis-induced ALI.

The circulating RNA concentration is a prognostic indicator of sepsis severity, and the plasma levels of host miRNAs (miR-210) in septic mice were markedly elevated^15^. The expression of miR-210 was significantly increased in plasma samples from patients with sepsis-induced acute kidney injury, while distinct reductions were detected in survivors of sepsis-induced acute kidney injury^16^. Our results suggest that the expression of miR-210-3p was increased in the plasma of septic patients and septic mice. We also observed that the expression of miR-210-3p was significantly upregulated in the lung tissue of septic mice, suggesting that miR-210-3p may play a role in regulating sepsis-induced ALI.

Sepsis can result in massive increases in EV production and release into the plasma associated with inflammation^21^. EVs can transfer receptors, proteins, and cytokines to stimulate and carry miRNAs hat alter gene expression and protein production at the molecular level^22^. In our study, 20 µg of plasma EVs were cultured with three types of lung tissue cells, (macrophages (THP cells), lung epithelial cells (BEAS-2B cells), and pulmonary microvascular endothelial cells (HLMVECs)). Our data demonstrated that septic-EVs increased the expression of inflammatory cytokines, reduced endothelial barrier function, increased permeability, and significantly diminished angiogenesis. These results suggested that septic-EVs promoted inflammation, apoptosis, and endothelial cell permeability to induce ALI.

Several studies have implicated miR-210-3p in autophagy and inflammation. The expression of miRNA-210 has been reported to be upregulated in dengue virus-infected C6/36 cells, which induces autophagy and lysozyme activity^23^. Furthermore, miRNA-210 is enriched in pathways associated with interferon signaling^24^. Moreover, Chao et al. concluded that miR-210-3p was increased in the blood following CLP^15^, while its role has not been functionally characterized. In our study, the overexpression of miR-210-3p promoted the expression of inflammatory factors, reduced cell viability, increased the apoptosis of BEAS-2B cells, and inhibited the function of the endothelial cell barrier and the formation of pulmonary microvascular endothelial cell tubules. These results emphasize the role of miR-210-3p in sepsis-induced inflammation, apoptosis, and endothelial cell dysfunction. However, the specific mechanism underlying this effect remains unknown. Therefore, miR-210-3p may be involved in sepsis-induced ALI by regulating other signaling pathways.

ATG7 is an autophagy-related gene that is essential for autophagosome biogenesis and has often been highlighted in the innate immune system^25^. ATG7 plays a crucial role in the inflammatory response to bacterial infection. Atg7 deficiency increased the production of IL-1β and enhanced inflammasome activation in *Pseudomonas*-induced sepsis^17^. Specific gene silencing of ATG7 in kidney proximal tubules worsened lipopolysaccharide (LPS)-induced AKI^19^. To determine the effect of miR-210-3p on ATG7 in sepsis-induced ALI, ATG7 was overexpressed in THP cells, BEAS-2b cells, and HLMVECs, followed by transfection with miR-210-3p. Our results suggested that miR-210-3p inhibited the expression of ATG7 and inhibited autophagy in the presence of ATG7 overexpression, leading to a marked reduction in miR-210-3p-induced damage to lung-related cells.

During the present study, EVs were cultured with macrophages (THP cells), lung epithelial cells (BEAS-2b cells), and HLMVECs. Our data suggested that septic-EVs augmented THP-1 macrophage inflammation, BEAS-2B cell apoptosis, and HLMVEC permeability while acting to suppress angiogenesis and cellular activity. These results indicate that septic-EVs promoted inflammation, apoptosis, and endothelial cell permeability to induce ALI. EVs are highly enriched and play dual roles in sepsis-induced ALI. Existing literature has suggested that EVs attenuate inflammation-induced lung injury^26^. EVs from endothelial progenitor cells diminish LPS-induced ALI via the encapsulation of miRNA−126^8^. However, peripheral circulating EVs shuttling miR-155 stimulate nuclear factor κB activation and induce the production of TNF-α and IL-6 to induce septic lung injury^9^. The dual roles of EVs in sepsis-induced ALI have been suggested to be attributed to the various functions of miRNAs. Our study demonstrated that septic-EVs induced ALI through the targeted inhibition of ATG7 and autophagy-related protein expression via miR-210-3p and that the inhibition of miR-210-3p may alleviate ALI. Autophagy is one of the innate immune defense mechanisms against microbial attack and is activated in the early stages of sepsis^6^. Autophagy has been reported to protect the lung^27^. Autophagy is mediated by ATG7^28^, which is the target gene of miR-210-3p. In our study, we identified that plasma EVs containing miR-210-3p were increased in CLP-induced septic mice, which further stimulated the secretion of inflammatory factors in lung tissues by inhibiting the effect of ATG7 on autophagy, promoting cell apoptosis, increasing tissue permeability, and ultimately causing ALI.

Taken together, the key findings of our study highlight that the plasma EV-mediated delivery of miR-210-3p targets ATG7 to regulate autophagy and inflammatory activation in sepsis-induced ALI. Targeting miR-210-3p is a potential promising therapeutic strategy for sepsis-induced ALI. During our study, we injected ad-anti-miR-210-3p to knock down miR-210-3p in mice, which may have lower efficacy and stability than the use of transgenic mice. However, transgenic mice should be used to further examine the roles of miR-210-3p in sepsis-induced ALI in the future.

## Supplementary information

Supplementary Tables
