# Supplementary material for: Plasma extracellular vesicle delivery of miR-210-3p by targeting ATG7 to promote sepsis-induced acute lung injury by regulating autophagy and activating inflammation
Source: Exp Mol Med. 2021 Jul 28;53(7):1180–91. doi: 10.1038/s12276-021-00651-6 (PMC8333093; doi:10.1038/s12276-021-00651-6)
Supplement: Supplementary file 1 — Supplementary Tables [file 12276_2021_651_MOESM1_ESM.docx]

**Table S1 qRT-PCR primer sequences**

| **Name** | **Sequence (5'-3')** |
| --- | --- |
| mmu-miR-210-3p RT-primer | GTCGTATCCAGTGCAGGGTCCGAGGTATTCGCACTGGATACGACTCAGCC |
| hsa-miR-210-3p RT-primer | GTCGTATCCAGTGCGTGTCGTGGAGTCGGCAATTGCACTGGATACGACTCAGCCG |
| mmu-miR-210-3p | F: CGCTGTGCGTGTGACAGC |
|  | R: AGTGCAGGGTCCGAGGTATT |
| has-miR-210-3p | F: AGGCTGTGCGTGTGACA |
|  | R: AGTGCGTGTCGTGGAGTCG |
| U6 (has- and mmu-) | F: TGCGGGTGCTCGCTTCGGCAGC |
|  | R: CCAGTGCAGGGTCCGAGGT |
| U6 RT primer | F: GTCGTATCCAGTGCAGGGTCCGAGGTATTCGCA |
|  | R: CTGGATACGACAAAATATGGAAC |
| mmu-ATG7 | F: TGACCTTCGCGGACCTAAAGA |
|  | R：CCCGGATTAGAGGGATGCTC |
| mmu-SQSTM1/p62 | F: GAACTCGCTATAAGTGCAGTGT |
|  | R: AGAGAAGCTATCAGAGAGGTGG |
| mmu-LC3B | F: GACCGCTGTAAGGAGGTG |
|  | R: CTTGACCAACTCGCTCATGTTA |
| has-LC3B | F: GATGTCCGACTTATTCGAGAGC |
|  | R: TTGAGCTGTAAGCGCCTTCTA |
| mmu-NLRP3 | F: TCTGACCTCTGTGCTCAAAACCAA |
|  | R: TGAGGTGAGGCTGCAGTTGTCTAAT |
| mmu-caspase1 | F: ACTGACTGGGACCCTCAAGTTTTGC |
|  | R: GGCAAGACGTGTACGAGTGGTTGTA |
| mmu-β-actin | F: GGCTGTATTCCCCTCCATCG |
|  | R: CCAGTTGGTAACAATGCCATGT |
| hsa-ATG7 | F: TGCTATCCTGCCCTCTGTCTT |
|  | R: TGCCTCCTTTCTGGTTCTTTT |
| hsa-SQSTM1/p62 | F: TGTGTAGCGTCTGCGAGGGAAA |
|  | R: AGTGTCCGTGTTTCACCTTCCG |
| hsa-β-actin | F: GTCATTCCAAATATGAGATGCGT |
|  | R: GCTATCACCTCCCCTGTGTG |
| has-IL-6 | F: CCTGAACCTTCCAAAGATGGC |
|  | R: TTCACCAGGCAAGTCTCCTCA |
| has-IL-1β | F: ATGATGGCTTATTACAGTGGCAA |
|  | R: GTCGGAGATTCGTAGCTGGA |
| has-TNF-α | F: CCAGGGACCTCTCTCTAATCA |
|  | R: TCAGCTTGAGGGTTTGCTAC |

**Table S2 Baseline charecteristics of healthy controls and septic patients.**

| **Index** | **Heathy controls** | **Septic patients** | ***p*** |
| --- | --- | --- | --- |
|  | **(n=30)** | **(n=55)** |  |
| Age | 52.17 ± 13.87 | 53.02 ± 15.88 | 0.806 |
| Male/Female | 16/14 | 28/27 | 0.830 |
| BMI(kg/m2) | 31.87 ± 13.62 | 33.04 ± 14.88 | 0.722 |
| APACHEII score/point | 13.53 ± 5.83 | 22.91 ± 9.86 | 0.001 |
| SOFA score/point | 4.87 ± 1.41 | 8.91 ± 4.08 | 0.001 |
| ALI risk factors [Case (%)] |  |  |  |
| Pneumonia | 0 | 15 (27.27%) |  |
| Blood transfusion | 0 | 3 (5.45%) |  |
| Pancreatitis | 0 | 2 (3.64%) |  |

Note: BMI (kg/m^2^): Body mass index, as weight/height squared (international unit kg/m^2^)
